# Effect of Comprehensive and Integrative Medical Services on Patients with Degenerative Lumbar Spinal Stenosis: A Pilot Study

**DOI:** 10.3390/medicina59122166

**Published:** 2023-12-14

**Authors:** Sang Bong Ko, Sang Gyu Kwak

**Affiliations:** 1 Department of Orthopaedic Surgery, School of Medicine, Daegu Catholic University, Daegu Catholic University Hospital, Daegu 42472, Republic of Korea; 2 Department of Medical Statistics, School of Medicine, Daegu Catholic University, Daegu 42472, Republic of Korea; sgkwak@cu.ac.kr

**Keywords:** spinal stenosis, nerve block, acupuncture therapy, comprehensive health care

## Abstract

*Background and Objectives*: Two types of medicinal systems are available in Korea: Western and oriental. These exist as separate services that independently provide medical care to patients. We determined the utility and benefits of compressive and integrated medical services (CIMS) comprising 12 sessions of acupuncture and healing programs over 6 weeks. *Methods and Methods*: In this two-group parallel single-center randomized controlled assessor-blinded trial, 25 participants were assigned to either the experimental (conventional medical treatment plus CIMS, n = 12) or control (conventional medical treatment, n = 13) group. Spinal nerve root block was performed on the compressed spinal nerve root (identified using magnetic resonance imaging) when no improvement was observed after the initial treatment. The experimental group received 12 cycles of acupuncture and manual therapy for 6 weeks; the control group received conventional medical treatment alone. *Results*: The average age of participants in the experimental and control groups was 70.73 ± 5.95 and 67.33 ± 8.89 years, respectively. There were no significant differences between the groups in terms of age, body mass index, Leeds Assessment of Neuropathic Symptoms and Signs, sex, and current medical history. We found high compliance for both programs (acupuncture and healing). On exclusion of between-group effects, the visual analog scale (VAS) score improved significantly over time (*p* = 0.045). Further, comparison of the groups after excluding the effects of visits revealed significantly lower VAS scores in the experimental group than in the control group (*p* = 0.000). *Conclusions*: Patients with degenerative lumbar spinal stenosis who mainly complain of radiating pain in the lower leg may benefit from CIMS after spinal nerve root block for ≤3 months after treatment. Our study findings suggest that this treatment improves spinal function and Oswestry Disability Index score. However, CIMS did not improve QoL.

## 1. Introduction

Degenerative lumbar spinal stenosis (DLSS) is one of the most common degenerative spinal diseases. This condition manifests as radiating lower leg pain (LLRP), low back pain (LBP), referred buttock pain, and neurogenic intermittent claudication (NIC). Both LLRP and referred buttock pain primarily affect the hip or lower-back area; however, they can also radiate to the thighs, calves, ankle joints, and soles. These symptoms are highly sensitive, accounting for 95% of nerve root symptoms, and they are mainly reported as stabbing, dullness, burning, electrifying, numbness, or worm-crawling sensations. Notably, gait disturbance is caused by hemodynamic disorders in the cauda equina region, and it is referred to as NIC as it is a representative lesion that reduces walking distance. Various studies have reported the effects of spinal manual therapy, physical therapy, massage, and numerous medications (anti-inflammatory drugs, muscle relaxants, calcium-channel blockers, etc.) used for relieving LLRP [1,2,3]. Epidurals and selective spinal nerve root block (SNRB) are commonly performed as minimally invasive treatments for LLRP; in particular, SNRB is used for pain relief because it involves injections of corticosteroids and local anesthetic into the compressed nerve root that causes radiating pain. Although the therapeutic effects of SNRB remain debatable, the prevailing opinion is that it is an effective short-term treatment option [4,5].

Two types of medicine are used in Korea: Western and oriental. Acupuncture is a part of oriental medicine that has been proven to be effective for LLRP in many studies [3,6,7,8]. Moreover, manual therapy involving joint mobilization, joint correction, soft-tissue mobilization, neural mobilization, and stabilization exercises have been reported to be effective for pain control and dysfunction in patients with DLSS [9]. These two approaches can be provided independently; however, there is a need for a cooperative treatment system incorporating Western and oriental medicine, as recently proposed by the Ministry of Health and Welfare of Korea in the third stage of the “Medicine-Oriental Medicine Cooperation Pilot Project”. This project aims to provide cooperative treatment services by combining Western and oriental medicines, and it has been in operation since 2017 [10]. Moreover, this project offers compressive and integrated medical services (CIMS), which include healing programs, regenerative medicine, and complementary alternative medicine.

The present study aimed to investigate the benefits of the use of additional CIMS in patients who required SNRB because of DLSS. Accordingly, conventional treatments, such as SNRB combined with medications, were administered to patients with DLSS who presented with LLRP as the predominant symptom. Patients who received additional CIMS (12 sessions of acupuncture and healing programs over 6 weeks) were compared with those who did not, and the pain-reducing effects, changes in health status, and safety of CIMS were evaluated.

## 2. Methods

### 2.1. Study Design and Patient Selection

This was a two-group parallel single-center randomized controlled assessor-blinded trial (registered at https://cris.nih.go.kr; KCT 0006036; date of trial registration: 29 March 2021). This study and its protocols were approved by the Institutional Review Board (approval Number: CR-20-205-L; date of approval: 26 November 2020). Written informed consent was obtained from all participants. A prospective single-blind randomized controlled trial was performed over a period of 3 months. Notably, this study is a pilot study for preliminary confirmation of the prudence of this study and clinical results and preparation of basic data prior to large-sample studies. For this study, patients were recruited in the experimental and control groups (those who received conventional medical treatment plus CIMS and those who received conventional medical treatment alone, respectively).

We recruited patients with DLSS (diagnosed by a spine physician) with a visual analog scale (VAS) score of ≥5 who were treated at a single institution between 1 April 2021, and 31 December 2021, and received SNRB. The inclusion and exclusion criteria are presented in Table 1. The diagnosis of DLSS was determined by the presence of LLRP, NIC, and LBP based on the following imaging findings [11]. Among the 10 parameters presented by Steuer et al. [12], an anteroposterior diameter of <10 mm, a cross-sectional area of <70 mm^2^ for the spinal canal, and the positive sedimentation sign of Barz et al. [13] were considered. In addition, as an objective assessment of LLRP, a score of ≥7 on the Leeds Assessment of Neurological Symptoms and Signs (LANSS) pain scale was considered to indicate LLRP.

### 2.2. Blinding and Randomization

A simple randomization procedure was used for this study. After generating a randomization code (allocation ratio; control group: experimental group = 1:1), the codes were sealed in opaque envelopes and drawn in order of enrollment to assign each participant to the control or experimental group. Single blinding was performed.

### 2.3. Epidemiological Chracteristics

Overall, 30 who met the inclusion criteria were included in this study. Among the 15 patients assigned to the experimental group, 1 was lost to follow-up because of a car accident, 1 withdrew consent, and 1 was judged to be inappropriate; thus, 12 patients (5 males, 7 females) were finally enrolled in the experimental group. Among the 15 patients assigned to the control group, 1 withdrew consent and 1 was judged to be inappropriate; thus, 13 patients (5 males, 8 females) were finally enrolled in the control group. This represented a dropout rate of 16.6%. The mean age of the participants was slightly higher in the experimental group, although the difference was not statistically significant. Body mass index (BMI), LANSS, sex, and existing medical history were not significantly different between the groups (Table 2).

### 2.4. Treatment Procedure

Limaprost 1T tid (Donga ST, Seoul, Republic of Korea) and Pregrabalin 75 mg bid (Pfizer, New York, NY, USA) as treatments for NIC and LLRP, respectively, were initially prescribed for 14 days to all participants. Participants who exhibited no improvement in the initial treatment underwent SNRB of the compressed spinal nerve root, which was identified via magnetic resonance imaging. The experimental group received 12 cycles of acupuncture and manual therapy over 6 weeks.

Acupoints were selected as proximal bilateral BL23, BL24, BL25, and Ex-B2 for LBP and unilateral GB30, BL40, and BL60 for LLRP in accordance with the Standard Korean Medicine Clinical Practice Guideline for DLSS [14]. Seventeen sterilized disposable stainless steel acupuncture needles (Dongbang Acupuncture Inc., Boryung, Republic of Korea), sized 0.25 × 40 mm or 0.30 × 60 mm, were inserted to a depth of approximately 15–50 mm depending on the acupoint using Gwanchim therapy. Subsequently, the needles manually stimulated using lifting-thrusting and rotation techniques to elicit a deqi sensation. Further, the acupoint needles were inserted for 25 ± 5 min and then removed. Acupuncture was performed by doctors of Korean medicine who were certified by the Korean Ministry of Health and Welfare and had >5 years of experience in the clinical field. Acupoints BL23, BL25, GB30, and GB31 were stimulated using 4 Hz electroacupuncture (ES-160; Ito Co., Ltd., Miki-City, Japan) for 25 min. Notably, the current was adjusted according to patient tolerance.

Manual therapy was performed for 30 min in each session by physical therapist using flexion distraction technique [1,2]. The therapist first identified the induration of the muscles and ligaments around the patient’s spine and then induced mobility and reduced pain by applying myofascial relaxation and massage or appropriate stretching therapy to relax the surrounding muscles and joints in order to achieve functional recovery.

### 2.5. Outcome Measurement

In this study, we investigated demographic factors (sex, age, height, and body weight), degree of LLRP (assessed by VAS), functional outcome (Oswestry Disability Index [ODI] and Roland–Morris Disability Questionnaire [RMDQ]), and quality of life (QoL) using the Short Form-36 (SF-36). VAS was used to determine the degree of pain reduction by evaluating the pain level perceived by the participant on a 10 cm scale (0, none; 10, most severe).

The SF-36 questionnaire was used to evaluate QoL and comprised 36 questions in 9 areas: 10 questions related to physical function, 4 related to the role limitation caused by physical health, 2 related to body pain, 5 related to general health, 3 related to role limitation caused by emotional problems, 4 related to energy and fatigue, 2 related to social functioning, 5 related to emotional well-being, and 1 related to change in health status. The first four areas determine the physical component summary (PCS) and the second four determine the mental component summary (MCS).

The short-term (2 weeks after treatment) and mid-term (6 and 12 weeks after treatment) treatment effects were evaluated and compared between the control and experimental groups. The compliance of the participants in the experimental group to acupuncture and manual therapy was calculated by dividing the number of acupuncture and manual therapy treatments by 12 (total times) and multiplying by 100.

### 2.6. Statistical Analysis

IBM SPSS Win. Ver. 19.0 was used for statistical analysis of the data, with the significance level set at 5%. A two-sided test was used for all the tests. Descriptive statistical analyses were performed for the demographic characteristics, and the means and standard deviations or frequencies and percentages (for quantitative and qualitative data, respectively) were summarized. The homogeneity of demographic and clinical characteristics was tested according to the group using the two-sample *t*-test or Mann–Whitney U-test for quantitative data, depending on normality. The chi-square test was performed for the qualitative data. Repeated-measures two-factor analysis was performed to determine differences in VAS, SF-36, ODI, and RMDQ scores between the groups at specific time points (before treatment and after 2, 6, and 12 weeks of treatment) and between time points within groups (interaction, interaction). Multiple comparisons were performed using the contrast method based on Bonferroni correction. Adverse event evaluation was performed for participants who underwent acupuncture and a healing program at least once. The chi-square test was used to compare the number of occurrences of adverse events related to acupuncture and healing programs between the groups, and the proportion of participants who experienced ≥1 adverse events was calculated.

## 3. Results

### 3.1. Compliance of the Experimental Group

All 12 participants exhibited 100% compliance with acupuncture treatment. For massage treatment, 11 participants exhibited 100% compliance and 1 exhibited 92% compliance.

### 3.2. VAS

**Figure 1:**
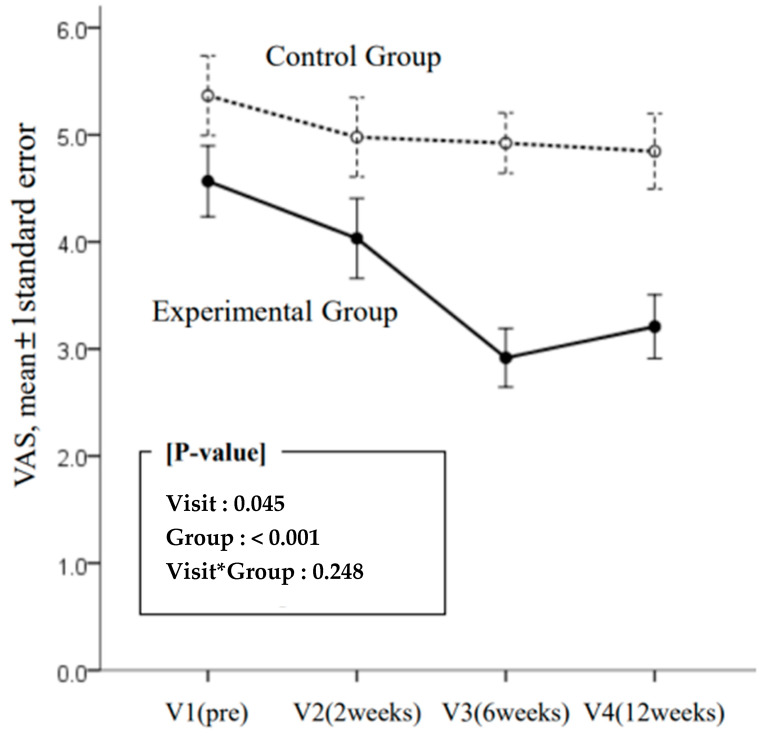
Results on visual analog scale by four visits (baseline; 2, 6, and 12 weeks) and two groups (experimental and control).

The VAS score (Table 3) revealed that VAS improved significantly over time and was significantly lower in the experimental group than in the control group. The change over time was not significantly different between the groups; however, the mean VAS score between points V2 and V3 increased by 0.02 and 0.66 in the control and experimental groups, respectively (Figure 1) and decreased by 0.66 in the experimental group with subsequent improvement of 0.29 from V3 to V4. This difference of 0.68 between the mean VAS score corresponds to 6.8% of the response range of the VAS measurement tool and 28.9% of the participants’ response range (2.35; minimum value: 2.92, maximum value: 5.25). Although there was no statistically significant difference due to the relatively high standard deviation value of the VAS, this difference of approximately 30% is considered a clinically significant change.

### 3.3. Functional Outcome and QoL

The RMDQ was not significantly different between of within the groups at any time point (Table 3). The change in RMDQ score was not significantly different between visits; moreover, the pattern of change within each or between groups was not significantly different over time. Significant improvement in the RMDQ scale changing from a score of 11.42 to 7.83 in the experimental group, while the control group remained stable. In the ODI scale the improvement is less marked (38.15 to 35.00) similar to that obtained in the control group. Moreover, the change in ODI was not significantly different between visit times or between groups over time; however, it was significantly higher in the control group than in the experimental group.

The change in PCS over time was not significantly different within or between groups, but the change at each time point was significantly different (Table 4). Notably, the change in MCS at each time point, between groups at each time point, and between groups over time was not significantly different. PCS, MCS, PF, EF and BP without variations in the final results between both groups. RPH, REP worse in the experimental group with respect to the control group. GH improves faster and is better maintained in the experimental group. RF, RPH, SF scores improve at 6 weeks then worsen at 12 weeks in experimental group, not in control in RPH and SF. It seems that PCS, MCS and GH scores improve a lot initially (from 32.14 to 40.31; from 25.00 to 19.17 and from 20.42 to 33.75, respectively) and then improve more slowly, while the control group does progressively slower, although they finally have the same results.

## 4. Discussion

As the first-line treatment for DLSS, conservative treatment mainly involves short-term bed rest, medication (including anticonvulsants (such as pregabalin), anti-inflammatory drugs, muscle relaxants, prostaglandin E1 analogs, and antidepressants) use, physical therapy, use of braces, thermal therapy, ultrasound, manual therapy, electrical stimulation, and traction therapy [15,16,17]. Notably, in patients with DLSS, prostaglandins, gabapentin, methyl cobalamin, and calcitonin injection are recommended for the treatment of LLRP [18,19]. Selective SNRB is one of the various treatment options that do not involve the administration of drugs, and it has been shown to have a better effect than placebo [20]. There is a lack of evidence on the treatment of LLRP using physical or exercise therapy [21]; thus, SNRB is an important alternative treatment option for LLRP patients with DLSS who do not respond to treatment with medications.

A previous study reported that the administration of hyaluronic acid-carboxymethylcellulose (HA-CMC) instead of corticosteroids, such as lidocaine and bupivacaine, which are associated with numerous complications, for SNRB prolonged the period of pain improvement [22,23]. However, if these treatments fail to treat LLRP sufficiently, surgical decompression may be indicated.

There are many traditional medical and conservative treatments for DLSS in Northeast Asian countries, including Korea. Moreover, various studies have reported that acupuncture—the main tool in oriental medicine—is effective for DLSS [6,7,8,24]. Among the conservative treatments performed for DLSS, manual therapy is one of the most commonly used. The flexion distraction technique intensively exercises the apophyseal joint by distracting a specific area of the lumbar spine, which further releases the anterior and posterior longitudinal ligaments and rearranges the intervertebral disk. It also relaxes the posterior facet joint, which is the only active joint in the spine of the posterior lateral joint, and supports approximately 30% of the load applied to the spine. This restores physiological movement of the spinal joint and alleviates pain. This technique has been shown to be effective for the treatment of sciatica and back pain [1,2]; therefore, it could be beneficial for LLRP in the context of DLSS as the treatment focuses on controlling the pain in such patients. As the level of pain can vary considerably, there is a need for diversity to ensure that the treatment can be tailored according to the degree of pain. Therefore, the present aimed to clarify the benefits of CIMS used in combination with conventional treatments for LLRP to broaden the treatment options.

The key findings of this study are as follows. First, CIMS combined with conventional medical treatment provides clinically superior effects in terms of pain control in patients with DLSS. Second, when CIMS was added to the conventional treatment for DLSS, pain control superiority persisted until 6 weeks of the cessation of treatment. Third, CIMS used in the present study included 12 sessions of oriental medical treatment and healing programs over 6 weeks, which were very well adhered to by the patients. In addition, the safety of this approach was confirmed because no adverse reactions were observed in any participant. Most previous studies on acupuncture treatment for LLRP in patients with DLSS have investigated the short-term effects (i.e., during and immediately after treatment), but they have reported persistence of effects even after cessation of treatment. Further, the improvement in pain assessment by VAS in patients in the experimental group that has been maintained over time. A significant improvement in RMDQ was also obtained with respect to the control and with respect to the start of treatment, although not significant, probably due to the number of patients. In the results of the ODI scale assessment, which evaluates more severe disability situations, the differences are similar, although significant between the groups.

The present study confirmed that additional CIMS relieves pain more effectively than conventional medical treatment alone. However, although this effect persisted even after the treatment was concluded, the magnitude of the effect gradually decreased after the cessation of treatment. In contrast, the evaluation index indicates that the effects of conventional medical treatment persist over time, indicating a disadvantage of CIMS in realizing the maximum effect at the time of treatment.

This study has several limitations. First, this was a preliminary clinical trial (pilot study), and more extensive studies are needed and warranted based on the results presented here. The results of this study can be used in the future to provide clinical information and basic data for large-scale clinical trials. It would be appropriate for them to justify or comment on the trends in the results obtained, even if they are not statistically significant. Post-analysis evaluation of the power of the change in VAS score revealed that the primary validity evaluation variable between the two groups had a high power (0.84). Second, this study only analyzed the short-term effects (6 weeks after termination) of CIMS. Third, this pilot study included a small number of participants; therefore, more significant differences can be confirmed if larger-scale studies are conducted.

## 5. Conclusions

Patients with DLSS who primarily complain of LLRP may benefit from CIMS after SNRB, as we have shown that it results in a steady improvement in pain for up to 3 months after treatment and improves assessment of spinal function. However, these effects did not improve the QoL. In conclusion, we believe that post-SNRB CIMS is a valid treatment option to improve pain and functional outcomes in patients with DLSS and LLRP. However, CIMS did not improve QoL.

## Figures and Tables

**Table 1:** Inclusion and Exclusion Criteria.

Inclusion Criteria	
1	Patients under 80 years of age diagnosed with DLSS by clinical symptoms and magnetic resonance imaging (MRI).
2	Patients with LLRP as the main symptom (LANSS > 7).
3	Patients complaining of LLRP that require SNRB (VAS > 5).
4	Subjects who voluntarily consented to written consent.
Exclusion Criteria	
1	Extreme age patients, under 20 years of age and over 80 years of age
2	Pregnant patient
3	Patients with secondary gain (industrial accident, auto insurance, etc.)
4	Patients with serious comorbidities
5	Patients with contraindications to the drug administered
6	Patients participating in an interventional study during the study period
7	Patients with cancer pain due to primary or metastatic cancer of the spine
8	Patients who cannot communicate with questionnaires, etc.
9	A patient was excluded if he or she have fear or phobia from needles

DLSS—degenerative lumbar spinal stenosis; LLRP—lower leg radiating pain; LANSS—Leeds Assessment of Neurological Symptoms and Signs; SNRB—selective nerve root block; VAS—visual analogue scale.

**Table 2:** Epidemiological Characteristics.

Variable		Experimental Group (n = 15)	Control Group (n = 15)	*p*-Value
Age		70.73 ± 5.95	67.33 ± 8.89	0.229
BMI		23.86 ± 2.68	24.71 ± 2.87	0.409
LANSS		11.87 ± 3.16	13.73 ± 2.63	0.090
Sex	Male	7 (46.7)	6 (40)	0.713
	Female	8 (53.3)	9 (60)	
Medical History	No	5 (33.3)	9 (60)	0.143
	Yes	10 (66.7)	6 (40)	
Detail	Eye	3	0	-
	Endocrine	3	0	
	Cardiovascular	2	2	
	Respiratory system	1	1	
	etc.	5	1	

Values were presented as the mean ± standard deviation or frequency (percent). BMI—body mass index; LANSS—Leeds Assessment of Neuropathic Symptoms and Signs.

**Table 3:** Comparison for VAS and functional outcomes between the study group and the control group.

Variable	Group	Visit, Mean ± SD				*p*-Value		
		V1 (Pre)	V2 (2 Weeks)	V3 (6 Weeks)	V4 (12 Weeks)	V	G	V*G
VAS	Exp	4.38 ± 1.21	3.58 ± 1.04	2.92 ± 0.95	3.21 ± 1.03	0.045	0.000	0.248
	Con	5.27 ± 1.48	4.90 ± 1.41	4.92 ± 1.02	4.85 ± 1.26			
RMDQ	Exp	11.42 ± 4.38	10.83 ± 5.29	10.08 ± 5.20	7.83 ± 3.01	0.219	0.171	0.520
	Con	12.62 ± 5.87	12.62 ± 4.98	11.08 ± 4.33	11.62 ± 3.55			
ODI	Exp	38.15 ± 10.84	34.44 ± 15.47	30.00 ± 8.40	35.00 ± 11.18	0.562	0.039	0.744
	Con	44.62 ± 21.74	42.22 ± 17.61	43.25 ± 13.29	41.03 ± 13.90			

Exp—experimental; Con—control; SD—standard deviation; VAS—visual analogue scale; RMDQ—Revised Roland–Morris Disability Scale; ODI—Oswestry Disability Index; V—visit; G—group.

**Table 4:** Comparison for QoL (quality of life) between the experimental group and the control group.

Variable	Group	Visit, Mean ± SD				*p*-Value		
		V1 (Pre)	V2 (2 Weeks)	V3 (6 Weeks)	V4 (12 Weeks)	V	G	V*G
PCS	Exp	32.14 ± 17.24	40.31 ± 18.64	43.75 ± 17.87	42.92 ± 17.85	0.017	0.503	0.841
	Con	29.66 ± 18.89	32.98 ± 20.92	38.94 ± 18.11	42.40 ± 17.37			
MCS	Exp	45.73 ± 24.83	52.4 ± 23.06	51.32 ± 21.36	52.00 ± 20.69	0.181	0.373	0.416
	Con	40.64 ± 18.66	39.45 ± 22.63	44.32 ± 18.78	55.35 ± 17.96			
PF	Exp	38.33 ± 19.23	49.17 ± 24.66	56.25 ± 15.09	42.08 ± 18.15	0.002	0.619	0.634
	Con	38.85 ± 23.2	46.15 ± 19.60	46.92 ± 19.21	41.54 ± 17.96			
RPH	Exp	25.00 ± 33.71	29.17 ± 38.19	41.67 ± 43.08	35.42 ± 44.54	0.394	0.924	0.693
	Con	23.08 ± 37.45	26.92 ± 38.81	30.77 ± 38.40	46.15 ± 40.63			
REP	Exp	44.44 ± 49.92	52.78 ± 43.71	50.00 ± 46.06	50.00 ± 43.81	0.393	0.390	0.446
	Con	23.08 ± 39.40	41.03 ± 38.86	33.33 ± 33.33	58.97 ± 41.17			
EF	Exp	42.08 ± 17.38	42.08 ± 17.12	37.92 ± 23.98	43.33 ± 13.54	0.666	0.553	0.711
	Con	37.31 ± 22.97	33.85 ± 19.60	39.62 ± 16.26	43.46 ± 15.33			
EWB	Exp	45.33 ± 28.66	54.33 ± 19.18	49.67 ± 23.66	56.33 ± 14.62	0.042	0.723	0.294
	Con	47.38 ± 20.90	40.62 ± 20.19	49.54 ± 21.26	60.31 ± 12.38			
SF	Exp	51.04 ± 26.89	60.42 ± 25.47	67.71 ± 14.56	58.33 ± 27.35	0.364	0.261	0.291
	Con	54.81 ± 26.29	42.31 ± 27.74	54.81 ± 27.74	58.65 ± 18.67			
BP	Exp	44.79 ± 20.52	49.17 ± 18.35	50.42 ± 22.71	56.25 ± 18.48	0.294	0.216	0.809
	Con	39.04 ± 24.65	36.92 ± 27.20	48.85 ± 14.92	50.00 ± 18.23			
GH	Exp	20.42 ± 22.51	33.75 ± 14.79	26.67 ± 16.14	37.92 ± 19.00	0.014	0.434	0.282
	Con	17.69 ± 17.75	21.92 ± 18.99	29.23 ± 18.47	31.92 ± 16.65			

Exp—experimental; C—control; SD—standard deviation; PCS—physical component score; MCS—mental component score; PF—physical functioning; RPH—role limitations due to physical health; REP—role limitations due to emotional problems; EF—energy/fatigue; EWB—emotional well-being; SF—social functioning; BP—body pain; GH—general health; V—visit; G—group.

## Data Availability

The datasets used and/or analyzed during the current study are available from the corresponding author on reasonable request.
